# The demographic features, clinicopathologic characteristics, treatment outcome and disease-specific prognostic factors of solitary fibrous tumor: a population-based analysis

**DOI:** 10.18632/oncotarget.6174

**Published:** 2015-10-19

**Authors:** Alimujiang Wushou, Yi-Zhou Jiang, Yi-Rong Liu, Zhi-Ming Shao

**Affiliations:** ^1^ Department of Breast Surgery, Key Laboratory of Breast Cancer in Shanghai, Fudan University Shanghai Cancer Center, Fudan University, Shanghai, China; ^2^ Department of Oncology, Shanghai Medical College, Fudan University, Shanghai, China

**Keywords:** Solitary fibrous tumor, SEER analysis

## Abstract

**Background:**

Solitary fibrous tumor's (SFT) demographic features, clinicopathologic characteristics, treatment outcome and disease-specific prognostic factors were unexplored comprehensively.

**Methods:**

SEER program was used to identify patients diagnosed with SFT from 1973 to 2012. Overall collected data were analyzed by using the SPSS 18.0.

**Results:**

In total, 804 cases were found including 613 cases with SFT-specific mortality and 801 patients were analyzed for overall survival (OS). The 3-year disease specific survival (DSS), 5-year DSS and 10-year DSS were 73.3%, 65.7% and 53.3%. The 3-year OS, 5-year OS and 10-year OS were 71.9%, 63.3% and 47.3%. In the multivariate survival analysis, the age > 51 years (hazard ratio [HR] = 1.851 for DSS, *P* = 0.024 and HR = 1.652 for OS, *P* = 0.033; Reference [Ref] ≤ 51 years for DSS and ≤ 53 years for OS), SEER stage metastasized tumor (HR = 4.269 for DSS, *P* = 0.000 and HR = 2.905 for OS, *P* = 0.028, Ref - localized + regional tumor), pathologic grade III + IV (HR = 2.734 for DSS, *P* = 0.001 and HR = 2.585 for OS, *P* = 0.000, Ref - grade I + II) were adversely associated with DSS and OS. In addition, surgery was favorably associated with DSS (HR = 0.217, *P* = 0.045, Ref - surgery + radiotherapy).

**Conclusions:**

The surgery was an independent prognostic factor for DSS. The patient's age, SEER stage and pathologic grade were SFT-specific independent prognostic indicators for DSS and OS.

## INTRODUCTION

Soft tissue sarcomas represent a heterogeneous group of mesenchymal tumors with variable clinical behavior and prognosis [1]. As a result, the treatment modalities of soft tissue sarcoma are increasingly subtype-specific. There are big challenges for the oncologist in treating patients with rare subtypes of soft tissue sarcoma, since adequate instructive data from clinical trials or even coherent studies are not available to guide rigorous evidence-based treatment [2]. Hemangiopericytoma was firstly named by Stout and Murray in 1942. It derives from pericytes around capillaries and postcapillary venules, thus, this tumor can be found in any sites of human body that contain capillaries [3]. The term “hemangiopericytoma” has been retired; tumors diagnosed as hemangiopericytoma in the past are now called solitary fibrous tumor (SFT) [4]. SFT is one of the most commonly misdiagnosed soft tissue sarcomas in clinic because of its unspecific initial clinical presentation. Moreover, accurate pathologic diagnosis of SFT is also a challenge, because up to 15% of all soft tissue sarcomas can have SFT-like pathologic characteristics [5].

Our previous study investigated head and neck (HN) SFTs and found that a treatment protocol emphasizing the surgical removal of the tumor as the first-line treatment, tumor size > 5.0cm, poor pathologic differentiation, deep tumor location and non-surgical treatment were independent adverse prognostic factors for HN SFTs [6, 7]. Currently, only case reports and retrospective series have been reported with relatively small of study population from variety of tumor location. Given the rarity of this tumor, information is still sporadic regarding their unique demographic, clinicopathologic, prognostic and biologic characteristics. Oncologists around the globe have limited management experience, resulting in heterogeneity of treatment paradigms and prognosis.

We speculate that a large nationwide population-based patient cohort may provide an opportunity to evaluate trends in demographic features, clinical presentation, patient characteristics, diagnosis, treatment modalities and prognostic factors of SFT. To better characterize unique demographic features, clinicopathologic characteristics, treatment outcome and prognostic factors of SFT, we carried out a comprehensive analysis of all patients with SFT registered in the Surveillance, Epidemiology, and End Results (SEER) public-access database collected from various geographic areas in the United States from 1973 to 2012.

## RESULTS

### The demographic and clinicopathologic characteristics of 613 SFT patients with disease-specific survival status

A total of 613 patients with diagnosis of SFT were found in the SEER database with SFT-specific mortality spanning from 1973 to 2012. The median follow-up time was 86.8 (range, 1-478) months. Gender distribution was almost equal between males and females (45.4% and 54.6%, respectively) and a peak incidence occurred during the fifth decade of life. Majority of SFT occurred in white people. About half of the tumors registration came from the Pacific coast. Approximately, one third of SFT cases originated from thoracic, abdominal and pelvic cavity (TAPC) and one-half of SFT cases were classified as SEER stage regional tumor. Surgery was the most common treatment modality and 49.4% of cases were treated with surgery alone. Most of the tumors were pathologically unclassified SFTs and they accounted for nearly 70% of cases. The baseline characteristics of 613 SFT patients are summarized in Table 1.

**Table 1:** The baseline characteristics of SFT patients in the SEER database

Demographic and clinicopathologic parameters		DSS				OS			
		Alive	Dead	Total	*P*-value	Alive	Dead	Total	*P*-value
**Gender**	Female	183	152	335	0.584	196	244	440	0.205
	Male	158	120	278		177	184	361	
**Age**	≤ 51 years	204	107	311	**0.000**	-	-	-	-
	> 51 years	137	165	302		-	-	-	-
	≤ 53 years	-	-	-	-	226	177	403	**0.000**
	> 53 years	-	-	-	-	147	251	398	
**Race**	White	267	229	496	0.178	293	361	654	**0.033**
	Black	31	19	50		34	37	71	
	Others	43	24	67		46	30	76	
**Marital status**	Single	100	48	148	**0.000**	104	73	177	**0.000**
	Married	189	154	343		211	235	446	
	Divorced	15	29	44		15	48	63	
	Others	37	41	78		43	72	115	
**CHSDA Region**	East	122	54	176	**0.000**	130	90	220	**0.000**
	Northern plain	29	63	92		31	97	128	
	Pacific coast	179	128	307		199	201	400	
	Southwest	11	27	38		14	39	53	
**Tumor Locations**	Central nerve system	97	60	157	**0.000**	105	91	196	**0.001**
	Head and neck	83	34	117		91	69	160	
	TAPC	100	109	209		109	174	283	
	Extremity	50	57	107		55	75	130	
	Other location	11	12	23		13	19	32	
**AJCC Stage**	I + II	26	0	26	**0.007**	33	0	33	**0.001**
	III + IV	12	4	16		16	6	22	
**SEER stage**	Localized	194	109	303	**0.000**	210	177	387	**0.000**
	Regional	72	58	130		77	74	151	
	Distant metastasized	31	47	78		36	75	111	
	Unstaged	37	48	85		43	88	131	
	Unclassified	7	10	17		7	14	21	
**Treatment**	Radiotherapy + surgery	10	10	20	**0.000**	10	15	25	**0.002**
	Surgery + radiotherapy	126	86	212		139	130	259	
	Surgery alone	181	122	303		194	217	411	
	Radiotherapy alone	7	14	21		7	22	29	
	OCM	2	3	5		3	4	7	
	Unknown	15	37	52		20	50	70	
**Pathologic grade**	Grade I	18	5	23	**0.014**	20	11	31	0.138
	Grade II	44	18	62		45	40	85	
	Grade III	19	18	37		21	24	45	
	Grade IV	37	30	67		41	42	83	
	Unclassified	223	201	424		246	311	557	

Abbreviations: SEER, surveillance, epidemiology, and end results; SFT, solitary fibrous tumor; DSS, disease specific survival; OS, overall survival; CHSDA, contract health service delivery areas; TAPC, thoracic, abdominal and pelvic cavity; OCM, Others combined modalities; AJCC, american joint committee on cancer.

### Disease specific survival (DSS) and Cox proportional hazards regression analysis

**Figure 1:**
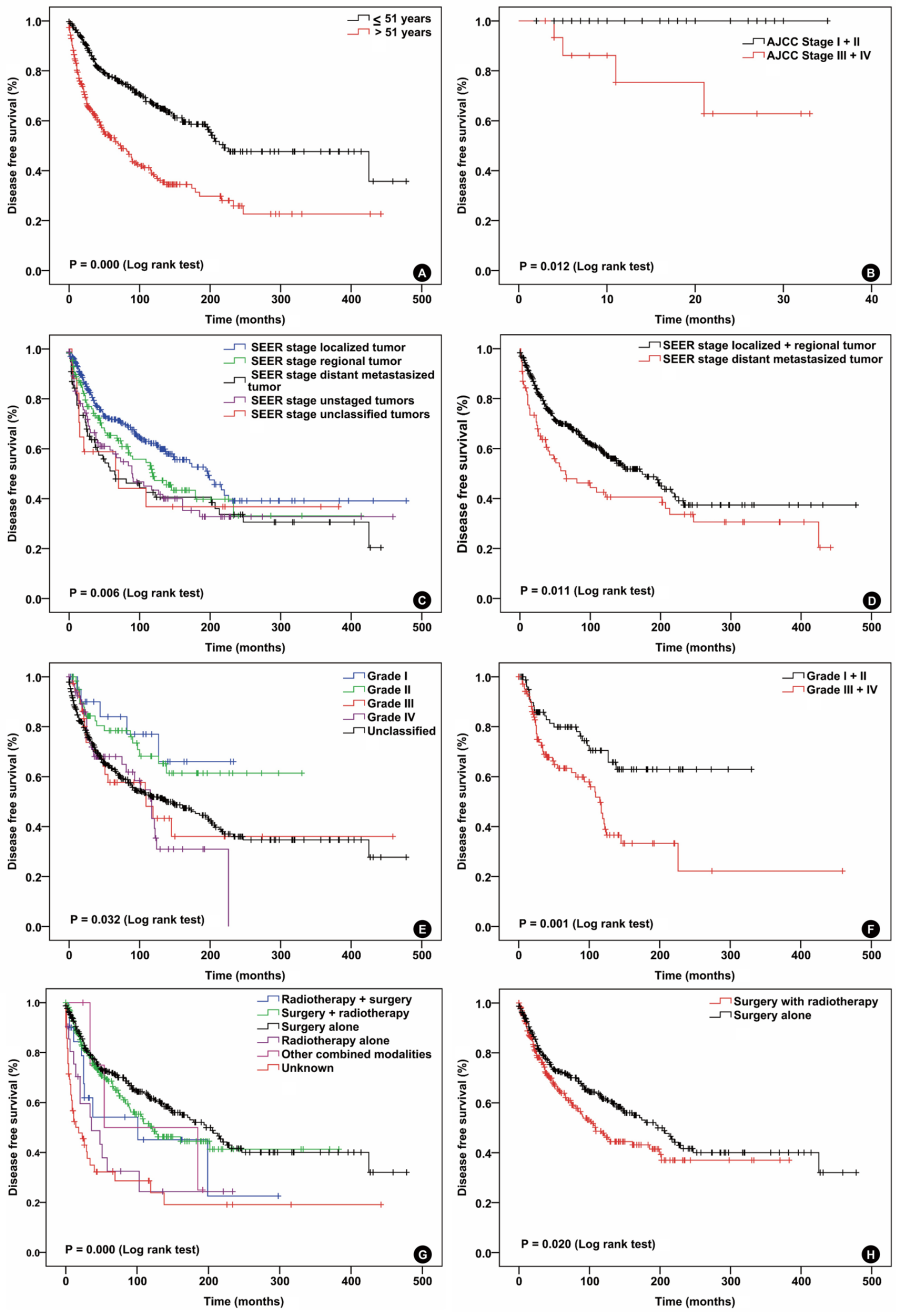
**Disease specific survival curves of patients with solitary fibrous tumor compared according to A.** age, **B.** AJCC stage, **C.** and **D.** SEER stage, **E.** and **F.** pathologic grade, **G.** and **H.** treatment modalities Log-rank test was used to compare curves, and significance (*P* value) is shown on each panel.

Kaplan-Meier analysis was performed for time-to-event analysis for DSS. The 3-year DSS, 5-year DSS, 10-year DSS, 15-year DSS were 73.3%, 65.7%, 53.3% and 45.9%, respectively. Significant DSS differences were found depending on age (*P* = 0.000), marital status (*P* = 0.000), SEER stage (*P* = 0.006), tumor location (*P* = 0.000), treatment modality (*P* = 0.000) and pathologic grade (*P* = 0.001) (Figure 1). A Cox proportional hazard model was performed to identify prognostic variables for DSS. In the univariate Cox regression analysis, the age >51 years (*P* = 0.000; HR=2.341; ≤51 years - as reference [Ref]), SEER stage distant metastasized tumor (*P* = 0.012; HR=1.532; localized + regional tumor - as Ref) and pathologic grade III + IV (*P* = 0.002; HR=2.210; grade I + II - as Ref) were associated with worse DSS. SFT from TAPC (*P* = 0.009; HR=0.412; central nerve system [CNS] - as Ref) and surgery alone (*P* = 0.000; HR=0.353; surgery with radiotherapy - as Ref) were associated with favorable DSS. In the multivariate Cox regression analysis, the age (*P* = 0.024; HR=1.851; ≤51 years - as Ref), SEER stage (*P* = 0.000; HR=4.269 for distant metastasized tumor; localized + regional tumor - as Ref), treatment modalities (P=0.045; HR=0.217 for surgery alone; surgery + RT - as Ref) and pathologic grade (*P* = 0.001; HR=2.734 for grade III + IV; grade I + II - as Ref) were independent prognostic variables for DSS. Details of the Cox proportional hazards regression analysis are shown in Tables 2 and 3.

**Table 2:** Univariate Cox regression analysis of characteristics associated with DSS and OS status

Parameters		DSS		OS	
		HR (95% CI)	*P*-value	HR (95% CI)	*P*-value
**Gender**	Female	1.00 Reference	0.786	1.00 Reference	0.834
	Male	1.034 (0.813-1.315)		0.980 (0.809-1.186)	
**Age**	≤ 51 years	1.00 Reference	**0.000**	1.00 Reference	**0.000**
	> 51 years	2.341 (1.829-2.996)		2.253 (1.850-2.742)	
**Race**	White	1.00 Reference		1.00 Reference	
	Black	1.353 (0.888-2.061)	0.159	1.430 (0.985-2.075)	0.060
	Others	1.317 (0.721-2.406)	0.370	1.662 (1.026-2.691)	**0.039**
**Marital status**	Married	1.00 Reference		1.00 Reference	
	Other status	1.006 (0.970-1.280)	0.964	1.049 (0.867-1.269)	0.626
**CHSDA Region**	East	1.00 Reference		1.00 Reference	
	Northern plain	0.411 (0.258-0.654)	**0.000**	0.628 (0.431-0.914)	**0.015**
	Pacific coast	0.925 (0.589-1.453)	0.736	0.996 (0.687-1.445)	0.985
	Southwest	0.570 (0.376-0.864)	**0.008**	0.761 (0.540-1.073)	0.119
**Locations**	Central nerve system	1.00 Reference		1.00 Reference	
	Head and neck	0.537 (0.288-1.001)	0.050	0.579 (0.353-0.950)	**0.031**
	TAPC	0.412 (0.213-0.798)	**0.009**	0.493 (0.296-0.822)	**0.007**
	Extremity	0.789 (0.434-1.435)	0.438	0.755 (0.470-1.215)	0.248
	Other location	0.800 (0.429-1.493)	0.484	0.696 (0.420-1.154)	0.160
**AJCC Stage**	Stage I + II	1.00 Reference	0.271	1.00 Reference	0.182 0.160
	Stage III + IV	11.035 (0.024-549.7)		0.010 (0.000-8.658)	
**SEER stage**	Localized + regional	1.00 Reference	**0.012**	1.00 Reference	**0.028**
	Distant metastasized	1.532 (1.100-2.134)		1.343 (1.032-1.749)	
**Treatment**	Radiotherapy + surgery	1.00 Reference		1.00 Reference	
	Surgery + radiotherapy	0.489 (0.242-0.992)	**0.047**	0.450 (0.252-0.803)	**0.007**
	Surgery alone	0.353 (0.237-0.526)	**0.000**	0.365 (0.261-0.508)	**0.000**
	Radiotherapy alone	0.294 (0.200-0.431)	**0.000**	0.333 (0.244-0.454)	**0.000**
	OCM	0.742 (0.398-1.384)	0.348	0.751 (0.454-1.242)	0.264
	Unknown	0.436 (0.134-1.422)	0.169	0.361 (0.130-1.000)	0.050
**Treatment**	Surgery with radiotherapy	1.00 Reference	**0.021**	1.00 Reference	0.053
	Surgery alone	0.738(0.570-0.955)		1.226 (0.997-1.506)	
**Pathologic grade**	Grade I + II	1.00 Reference	**0.002**	1.00 Reference	**0.005**
	Grade III + IV	2.210 (1.338-3.649)		1.694 (1.169-2.455)	

Abbreviations: SEER, surveillance, epidemiology, and end results; SFT, solitary fibrous tumor; DSS, disease specific survival; OS, overall survival; HR, hazard ratio; CI, confidence interval; CHSDA, contract health service delivery areas; TAPC, thoracic, abdominal and pelvic cavity; OCM, Others combined modalities; AJCC, American joint committee on cancer.

**Table 3:** Multivariate Cox regression analysis of characteristics associated with DSS and OS status

Parameters		DSS		OS	
		HR (95% CI)	*P*-value	HR (95% CI)	*P*-value
Age	≤ 51 years	1.00 Reference	0.024	1.00 Reference	0.033
	> 51 years	1.851 (1.083-3.165)		1.652 (1.042-2.618)	
SEER stage	Localized + regional	1.00 Reference	0.000	1.00 Reference	0.028
	Metastasized	4.269 (2.144-8.499)		2.905 (1.673-5.043)	
Treatment modalities	Radiotherapy + surgery	1.00 Reference		1.00 Reference	
	Surgery + radiotherapy	0.493 (0.129-0.719)	0.412	0.880 (0.170-4.572)	0.879
	Surgery alone	0.217 (0.156-0.747)	0.045	0.811 (0.158-2.364)	0.476
	Radiotherapy alone	0.229 (0.375-2.994)	0.055	0.586 (0.154-2.225)	0.432
Pathologic grade	Grade I + II	1.00 Reference	0.001	1.00 Reference	0.000
	Grade III + IV	2.734 (1.517-4.925)		2.585 (1.556-4.284)	

Abbreviations: DSS, disease specific survival; OS, overall survival; HR, hazard ratio; CI, confidence interval; SEER, surveillance, epidemiology, and end results.

### The demographic and clinicopathologic characteristics of 801 SFT patients with overall survival (OS) status

From 1973 to 2012, there were 804 consecutive registered patients with SFT in the SEER database. Three patients were excluded for survival analysis, due to lack of treatment and follow-up data. The 801 patients included 361 male patients and 440 female patients, with a male-to-female ratio of 0.82 : 1. Their ages ranged from 1 month to 85+ years, with a median of 53 years (Supplementary Figure 1). More than 80% of SFT cases occurred in white people and only 8.8% in black people. Half of the cases came from the Pacific coast, 220 cases from east, 128 cases from the northern plains and 53 cases from southwest. In total, 35.3% of tumors originated from TAPC, 24.5% from CNS, 19.9% from HN region and 16.2% from extremity. According to SEER stage classification, 48.3%, 18.9% and 13.9% patients were classified as localized, regional and distant metastasized SFTs. 87.6% patients treated with surgery included treatment modalities. Only 244 SFT patients had defined pathologic grade data. The median follow-up period was 85.6 (range, 1-478) months. The baseline characteristics of 801 SFT patients with OS status are presented in Table 1.

### OS and Cox proportional hazards regression analysis

**Figure 2:**
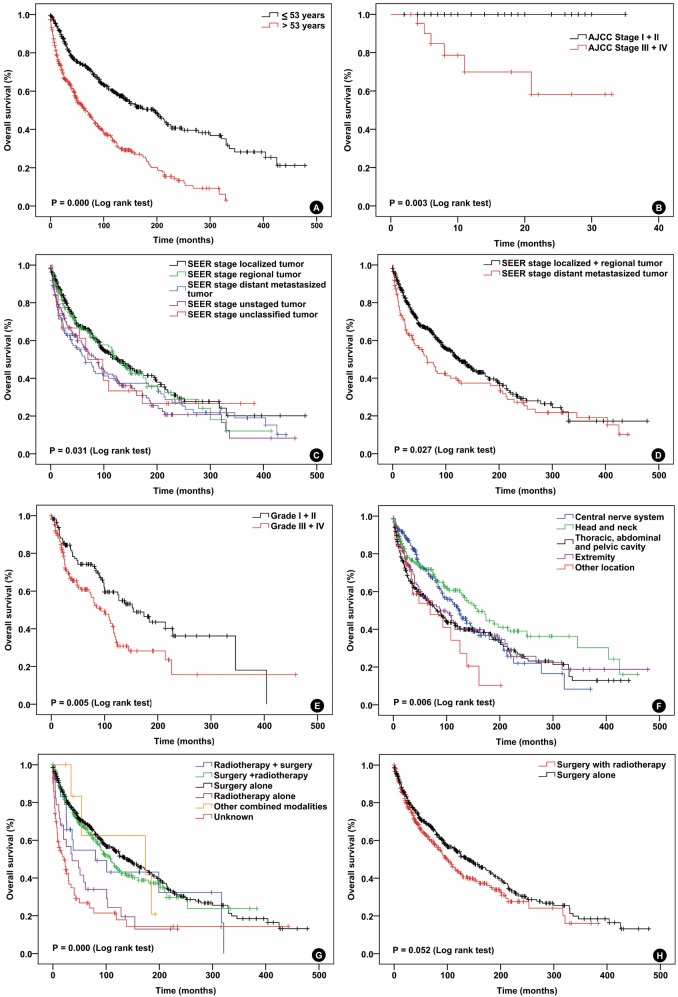
**Overall survival curves of patients with solitary fibrous tumor compared according to A.** age, **B.** AJCC stage, **C.** and **D.** SEER stage, **E.** pathologic grade, **F.** tumor location **G.** and **H.** treatment modalities Log-rank test was used to compare curves, and significance (*P* value) is shown on each panel.

The 3-year OS, 5-year OS, 10-year OS and 15-year OS rates were 71.9%, 63.3%, 47.3% and 38%, respectively. Significant OS differences were identified depending on age (P = 0.000), marital status (*P* = 0.000), CHSDA region (*P* = 0.006), tumor location (P = 0.006), AJCC stage (*P* = 0.003), SEER stage (*P* = 0.027), treatment modality (*P* = 0.000) and pathologic grade (*P* = 0.005) (Figure 2 and Supplementary Figure 2). Univariate and multivariate survival analysis utilizing the Cox regression model were performed on the 801 SFT patients. In the univariate Cox regression analysis, the age >53 years (*P* = 0.000; HR=2.253, ≤53 years - as Ref), distant metastasized tumor (*P* = 0.028; HR=1.343; localized + regional tumor - as Ref) and pathologic grade III + IV (*P* = 0.005; HR=1.694; Grade I + II - as Ref) were adversely associated with OS. SFT from TAPC (*P* = 0.007, HR=0.493; CNS- as Ref), surgery (*P* = 0.000, HR=0.365; RT + surgery - as Ref) were favorably associated with OS. The age (*P* = 0.033, HR=1.652 for >53 years; ≤53 years - as Ref), SEER stage (*P* = 0.028; HR=2.905 for distant metastasized tumor; localized + regional tumor - as Ref) and pathologic grade (*P* = 0.000; HR=2.585 for grade III + IV; grade I + II - as Ref) were independent prognostic variables for OS (Tables 2 and 3).

## DISCUSSION

According to the current investigation, incidence rate in gender is almost equal for DSS and slightly higher in female for OS. There is no statistically significant predominance in any gender. In survival analysis, remarkable difference was neither identified in DSS, nor in OS (*P* = 0.786 and *P* = 0.833, respectively, Supplementary Figure 2). In most of the previously published case series, the typical age at presentation ranged from the third to the fifth decades [8-10]. In this series, SFT most frequently occurs during the fifth decades of life (Supplementary Figure 2). The majority of investigation about SFT are based on single-center experience and mostly include no more than 30~50 cases. Given their small sample size, those investigations are often not sufficiently powerful to detect small differences in survival analysis according to common demographic factors such as age, gender and race. In our current investigation, patient's age, gender and race were analyzed as categorical variables in both univariate and multivariate survival analyses. The results demonstrate that apart from the age, no survival difference in DSS or OS was found to be associated with race or gender (Table 2 and Supplementary Figure 2).

Up to date, more than two thousand studies are available about SFT in the PubMed. SFT is reported from a variety of tumor locations. In accordance with previous reports, SFT occurred at any age-period and any site of the body in present series (Supplementary Figure 1). To better characterize, we categorized the tumor location into five groups (CNS, HN, TAPC, extremity and other location), although there are many other possibilities to categorize the tumor location. In this categorization, significant survival differences were found, where HN and TAPC SFTs were favorably associated with DSS (Ref - CNS) in univariate survival analysis. However, tumor location was not an independent prognostic indicator in multivariate survival analysis for neither DSS nor OS. Well consisting with our current results, previously, investigators found survival difference between CNS and extra-CNS site SFTs, and no association was found between the primary tumor site and survival [11].

The TNM/AJCC staging system plays important roles and it helps the oncologist in making a treatment protocol and evaluating a prognosis of cancer [12]. In this series, one of the most incomplete data was TNM/AJCC staging information and only 55 patients' data were available. However, important findings emerged. There was a significant survival difference in AJCC stage I + II versus AJCC stage III + IV. The small number of patients with AJCC stage in the Cox model did not permit a multivariate survival analysis for independent prognostic factor. In our previous report, we also failed to perform the AJCC stage in multivariate survival analysis of HN SFT due to the incomplete staging data. As far as we know, there aren't any report regarding TNM/AJCC stage identified as an independent prognostic variable for SFT. Nevertheless, detailed SEER stage data was available in this study and it was an independent prognostic indicator not only for DSS but also for OS.

Whether the SFT patients can benefit from RT is most widely investigated hot topic, especially in the CNS SFTs [13-17]. One of the main interests of this study is to hopefully confirm the role of RT in SFT treatment. The RT for SFT has been widely used alone or combined with surgery as part of the management algorithm for several decades. Until now, the role of RT in SFT treatment is still controversial. Lately, investigators analyzed 227 CNS SFT cases in the SEER database during the years of 2000-2009 and confirmed a survival benefit for patients treated with surgery in combination with adjuvant RT, while the effect was not appreciated with surgery alone. In another series, investigators found that treatment protocol of combined adjuvant RT with surgery seemed to hinder tumor progression, but had no effect on OS [18]. The addition of adjuvant RT to surgery improved the local recurrence, but did not increase overall survival of patients with HN SFTs [7, 19]. Owing to lack of data about recurrence, we were unable to confirm whether the RT could decrease the local recurrence in current series. We categorized treatment modalities including surgery and RT as surgery with RT group and compared its treatment outcome with surgery alone. The results demonstrate that surgery alone group had higher DSS and OS, and statistical significance was only found in DSS (Figure 1H and Figure 2H). It is noteworthy that mostly advanced stage SFT patients received RT pre- or postoperatively, and RT was added to early stage SFT once coming across common adverse prognostic factors after surgery such as high pathologic grade and unconfirmed surgical margins [6, 7, 10]. As retrospective study, it is almost impossible to make a standard survival comparison between patients treated with surgery alone and surgery with RT. Because those patients did not even have similar TNM/AJCC stage. Therefore, it is difficult to draw a firm conclusion that SFT patients cannot benefit from RT. Multi-center prospective controlled clinical studies are necessary to further confirm the role of RT in SFT treatment.

We acknowledge the limitations that come with our investigation. In the SEER database, data on important factors such as TNM/AJCC stage and pathologic grade are incomplete. Establishment of the role of chemotherapy is another important issue. A notable limitation of the SEER database is that there is no chemotherapy information available for analysis. In addition, not all patients have complete information.

## CONCLUSIONS

In summary, to the best of our knowledge, the present comprehensive analysis of the overall 804 cases from SEER database is the first of its kind to clearly define the demographic features, clinicopathologic characteristics, treatment outcome and prognostic factors of patients with SFT, and is also the largest SFT series to date. Despite the above limitations, for the first time we found that the age >51 years (Ref- ≤51 years, ≤53 years for OS), SEER stage distant metastasized tumor (Ref- localized + regional tumor) and high pathologic grade III + IV (Ref- Grade I + II) were independent averse prognostic indicators for DSS and OS. Regarding the treatment, the surgery was an independent favorable prognosis factor for DSS (Ref- RT + surgery).

## MATERIAL AND METHODS

### Date extraction

SEER*Stat software from the National Cancer Institute (Surveillance Research Program, National Cancer Institute SEER*Stat software, http://www.seer.cancer.gov/seerstat, Version 8.2.1) was applied for data extraction. International Classification of Diseases for Oncology (ICD-O-3) codes for hemangiopericytoma (9150) was used for identification of cases with a diagnosis of SFT registered in the SEER database.

### Variable selection and statistical analysis

The study variables included gender, age, race, marital status, contract health service delivery areas (CHSDA) region, primary tumor location, SEER stage, American joint committee on cancer (AJCC) stage, pathologic grade, treatment modalities, follow-up time and outcome status. Not all of the cases that we identified contained all these data. Collected data were analyzed using the software of the Statistical Package for Social Sciences, version 18.0, for Windows (SPSS, Chicago, IL). Differences in the numerical variables were assessed using the Student's test or non-parametric Wilcoxon test. The chi square test or Fisher exact test for categorical variables was used for two group comparisons of parameters. The survival curves were generated using the Kaplan-Meier method, and the log-rank test was performed to evaluate the survival difference. Adjusted hazard ratios (HRs) along with 95% confidence intervals (CIs) were calculated using the Cox proportional hazards regression model. When the P value was < 0.05, the difference was regarded as statistically significant. All statistical tests were two tailed.

## SUPPLEMENTARY MATERIAL FIGURES


