# Supplementary material for: The demographic features, clinicopathologic characteristics, treatment outcome and disease-specific prognostic factors of solitary fibrous tumor: a population-based analysis
Source: Oncotarget. 2015 Oct 19;6(39):41875–83. doi: 10.18632/oncotarget.6174 (PMC4747195; doi:10.18632/oncotarget.6174)
Supplement: Supplementary file 1 [file oncotarget-06-41875-s001.pdf]

The demographic features, clinicopathologic characteristics, treatment outcome and disease-specific prognostic factors of solitary fibrous tumor: a population-based analysis

Supplementary Material

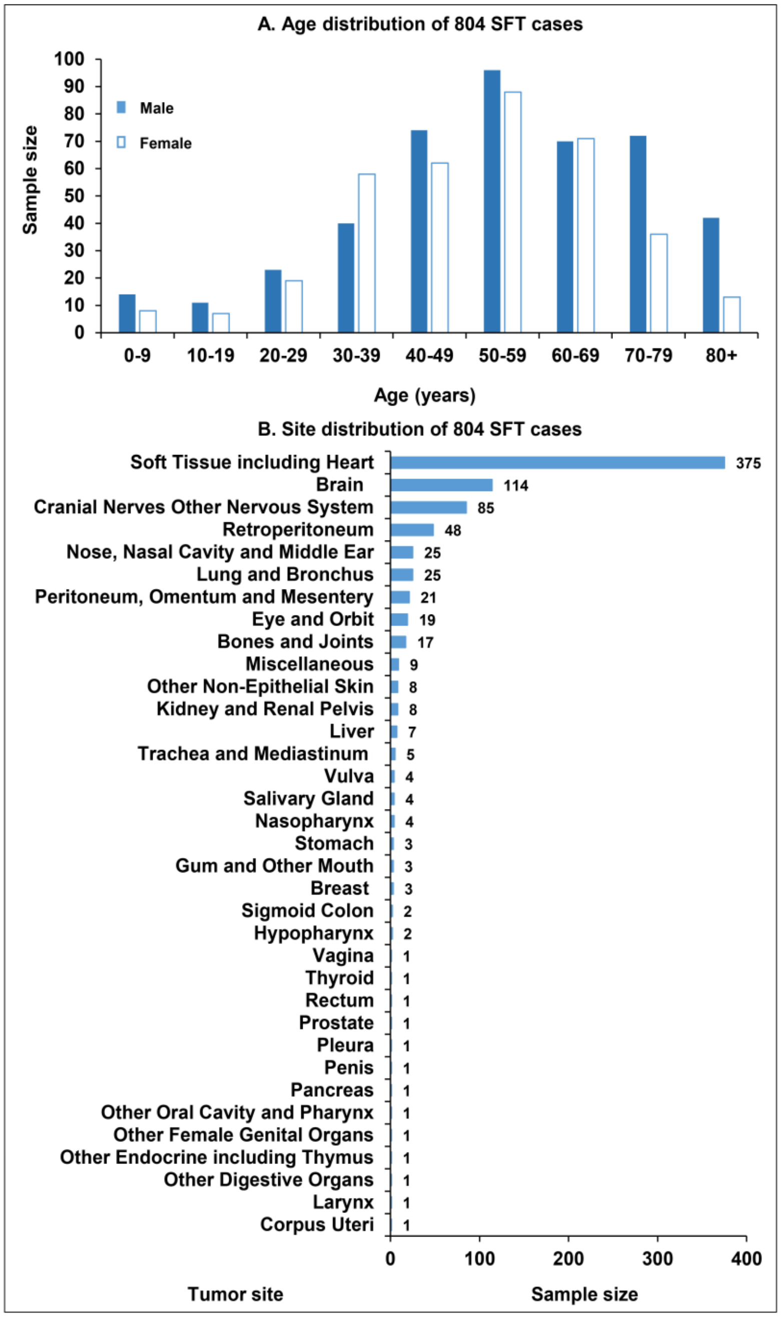

Supplementary figure 1: Age and primary tumor site distribution of 804 patients with solitary fibrous tumor.

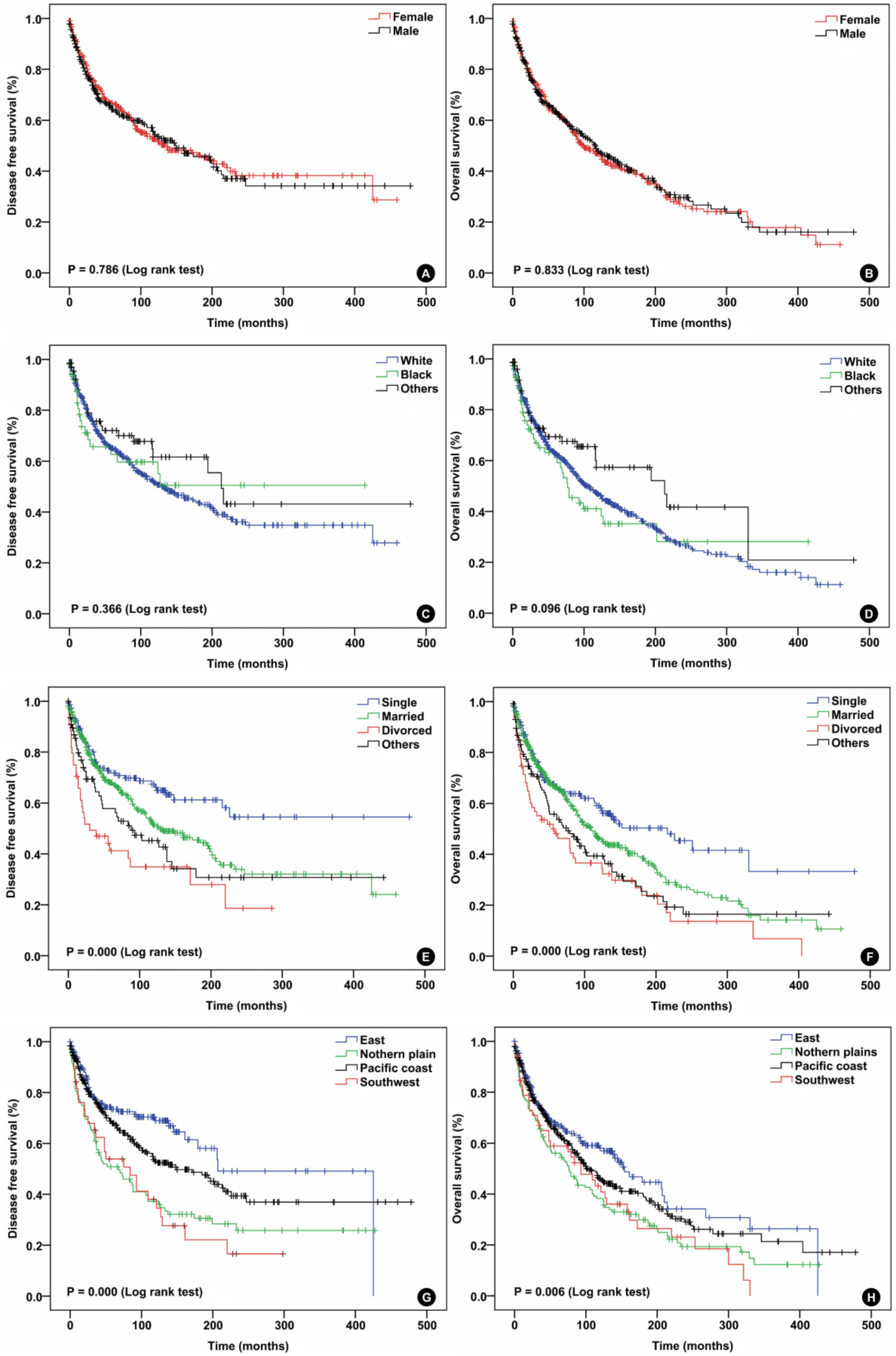

Female
Male
Disease free survival (%)
P = 0.786 (Log rank test)
A
Time (months)
Female
Male
Overall survival (%)
P = 0.833 (Log rank test)
B
Time (months)
White
Black
Others
Disease free survival (%)
P = 0.366 (Log rank test)
C
Time (months)
White
Black
Others
Overall survival (%)
P = 0.096 (Log rank test)
D
Time (months)
Single
Married
Divorced
Others
Disease free survival (%)
P = 0.000 (Log rank test)
E
Time (months)
Single
Married
Divorced
Others
Overall survival (%)
P = 0.000 (Log rank test)
F
Time (months)
East
Nothern plain
Pacific coast
Southwest
Disease free survival (%)
P = 0.000 (Log rank test)
G
Time (months)
East
Nothern plains
Pacific coast
Southwest
Overall survival (%)
P = 0.006 (Log rank test)
H
Time (months)

Supplementary figure 2: Disease free and overall survival curves of patients with solitary fibrous tumor compared according to (A) and (B) gender, (C) and (D) race, (E) and (F) marital status and (G) and (H) contract health service delivery areas. Log-rank test was used to compare curves, and significance (*P* value) is shown on each panel.
